# A gene-protein assay for human epidermal growth factor receptor 2 (HER2): brightfield tricolor visualization of HER2 protein, the *HER2* gene, and chromosome 17 centromere (CEN17) in formalin-fixed, paraffin-embedded breast cancer tissue sections

**DOI:** 10.1186/1746-1596-7-60

**Published:** 2012-05-30

**Authors:** Hiroaki Nitta, Brian D Kelly, Mary Padilla, Nikolaus Wick, Patrick Brunhoeber, Isaac Bai, Shalini Singh, Jim Ranger-Moore, Chris Bieniarz, Hitoshi Tsuda, Thomas M Grogan

**Affiliations:** 1 Medical Innovation, Ventana Medical Systems, Inc., Tucson, AZ, USA; 2 Technology and Applied Research, Ventana Medical Systems, Inc., Tucson, AZ, USA; 3 Roche Tissue Diagnostics, Roche Diagnostics S.L., Barcelona, Spain; 4 Scientific Affairs, Ventana Medical Systems, Inc., Tucson, AZ, USA; 5 Medical Affairs, Ventana Medical Systems, Inc., Tucson, AZ, USA; 6 Biostatistics and Data Management, Ventana Medical Systems, Inc., Tucson, AZ, USA; 7 Department of Pathology, National Cancer Center Hospital, Tokyo, Japan

**Keywords:** Gene-protein assay, Dual color *in situ* hybridization, Immunohistochemistry, HER2, Breast cancer

## Abstract

**Background:**

The eligibility of breast cancer patients for human epidermal growth factor receptor 2 (HER2)-directed therapies is determined by the *HER2* gene amplification and/or HER2 protein overexpression status of the breast tumor as determined by *in situ* hybridization (ISH) or immunohistochemistry (IHC), respectively. Our objective was to combine the US Food and Drug Administration (FDA)-approved *HER2* & chromosome 17 centromere (CEN17) brightfield ISH (BISH) and HER2 IHC assays into a single automated HER2 gene-protein assay allowing simultaneous detection of all three targets in a single tissue section.

**Methods:**

The HER2 gene-protein assay was optimized using formalin-fixed, paraffin-embedded (FFPE) samples of the xenograft tumors MCF7 [HER2 negative (non-amplified gene, protein negative)] and Calu-3 [HER2 positive (amplified gene, protein positive)]. HER2 IHC was performed using a rabbit monoclonal anti-HER2 antibody (clone 4B5) and a conventional 3,3'-diaminobenzidine IHC detection. The *HER2* & CEN17 BISH signals were visualized using horseradish peroxidase-based silver and alkaline phosphatase-based red detection systems, respectively with a cocktail of 2,4-dinitrophenyl-labeled *HER2* and digoxigenin-labeled CEN17 probes. The performance of the gene-protein assay on tissue microarray slides containing 189 randomly selected FFPE clinical breast cancer tissue cores was compared to that of the separate HER2 IHC and *HER2* & CEN17 BISH assays.

**Results:**

HER2 protein detection was optimal when the HER2 IHC protocol was used before (rather than after) the BISH protocol. The sequential use of HER2 IHC and *HER2* & CEN17 BISH detection steps on FFPE xenograft tumor sections appropriately co-localized the HER2 protein, *HER2* gene, and CEN17 signals after mitigating the silver background staining by using a naphthol phosphate-containing hybridization buffer for the hybridization step. The HER2 protein and *HER2* gene status obtained using the multiplex HER2 gene-protein assay demonstrated high concordance with those obtained using the separate HER2 IHC and *HER2* & CEN17 BISH assays, respectively.

**Conclusions:**

We have developed a protocol that allows simultaneous visualization of the HER2 IHC and *HER2* & CEN17 BISH targets. This automated protocol facilitated the determination of HER2 protein and *HER2* gene status in randomly selected breast cancer samples, particularly in cases that were equivocal or exhibited tumor heterogeneity. The HER2 gene-protein assay produced results virtually equivalent to those of the single FDA-approved HER2 IHC and *HER2* & CEN17 BISH assays.

**Virtual slides:**

The virtual slides for this article can be found here: http://www.diagnosticpathology.diagnomx.eu/vs/2041964038705297

## Background

Breast cancer is the most common cause of cancer death in Europe and the second leading cause of cancer death in the United States. The oncogene *HER2*, which encodes human epidermal growth factor receptor 2 (HER2) protein, is amplified in 20–30% of breast cancer cases [1] and is the target of HER2-directed anti-cancer therapies. Trastuzumab (Herceptin; Genentech, South San Francisco, CA, USA), a humanized monoclonal anti-HER2 antibody, has therapeutic effects against *HER2* gene and/or HER2 protein positive breast cancers as an adjuvant therapy. The small molecule dually targeted drug Lapatinib (Tyverb/Tykerb; GlaxoSmithKline, London, United Kingdom), which inhibits the tyrosine kinase activities of the HER2 and of epidermal growth factor receptor (EGFR) proteins, is a first-line therapeutic agent against triple positive breast cancers (positive for HER2 protein, estrogen receptor, and progesterone receptor) and it is also used in treating breast cancers refractory to trastuzumab therapy. Several drugs are currently in Phase III clinical trials for treatment of HER2 positive breast cancer, including pertuzumab, neratinib, and afatinib.

The American Society of Clinical Oncology (ASCO) and the College of American Pathologists (CAP) have introduced guidelines for HER2 status assessments based on the level of HER2 protein overexpression determined by immunohistochemistry (IHC) and on the level of *HER2* gene amplification determined by *in situ* hybridization (ISH) on formalin-fixed, paraffin-embedded (FFPE) breast cancer tissue sections [2]. However, which of the two methods is superior for assessing the HER2 status of breast cancer patients is unclear.

The two HER2 IHC-based diagnostic tests for assessing HER2 protein expression that are approved by the US Food and Drug Administration (FDA) use either a rabbit polyclonal (DAKO, Glostrup, Denmark) or a rabbit monoclonal (Ventana Medical Systems, Inc., Tucson, AZ, USA) antibody against HER2 protein. The results of these tests are scored semi-quantitatively as either 0 (negative), 1+ (negative), 2+ (equivocal), or 3+ (positive) [2]. The four FDA-approved ISH diagnostic tests for quantifying *HER2* gene copy numbers are dual color fluorescence ISH (FISH) (Abbott Molecular, Illinois, USA), single color chromogenic ISH (CISH) (Invitrogen, California, USA), dual color brightfield *in situ* hybridization (BISH) (Ventana), and dual color CISH (Dako) assays. For the dual color assays, results are determined as the ratio of the *HER2* gene signal to the chromosome 17 centromere (CEN17) signal (negative: *HER2*/CEN17 < 1.8; equivocal: 1.8 ≤ *HER2*/CEN17 ≤ 2.2; positive: *HER2*/CEN17 > 2.2) [2]. The results of single color ISH assays are considered positive if they detect six or more *HER2* gene copies and negative if they detect fewer than six [3].

*HER2* gene status and HER2 protein expression are generally concordant in breast cancer [4]. However, discordance between HER2 IHC and *HER2* ISH assay results can be caused by various factors and is not uncommon. For example, variations in tissue processing protocols affect HER2 protein detection more than the *HER2* gene detection; thus ISH assays can be more accurate than IHC assays when the pre-analytical process is not standardized [4]. Tumor heterogeneity can also contribute to discordance between HER2 IHC and *HER2* ISH scoring [5]. The possibility that a breast cancer patient will receive an incorrect HER2 status assessment is decreased when both assays are used, particularly when the cases are equivocal [6].

The simultaneous brightfield detection of HER2 protein and *HER2* gene expression “HER2 gene-protein assay” in FFPE breast cancer tissue sections has been previously reported by three independent groups [7-9]. First, Downs-Kelly *et al.*[7] successfully combined an alkaline phosphatase (AP)-based fast red dye system for HER2 IHC with a horseradish peroxidase (HRP)-based silver deposition system for *HER2* ISH. Then, Ni *et al.*[8] combined a fast red dye system for HER2 IHC with an HRP-based 3,3′-diaminobenzidine (DAB) system for *HER2* ISH. In the most recent report of a HER2 gene-protein assay, Reisenbichler *et al.*[9] used DAB-based detection for both HER2 IHC and *HER2* ISH in a single color HER2 gene-protein assay. Co-localization of CEN17 was not included in any of these HER2 gene-protein assays, even though using the copy numbers of both the *HER2* gene and CEN17 is considered optimal in determining *HER2* gene status for possible anti-HER2 therapies [4]. Furthermore, the previously described HER2 gene-protein assays were performed with semi-automated protocols requiring some manual steps.

In this study, our objectives were: 1) to develop an automated HER2 gene-protein assay for simultaneous tricolor visualization of HER2 protein, the *HER2* gene, and CEN17 in FFPE xenograft tumors and clinical breast cancer cases and 2) to evaluate the performance of this assay in determining the HER2 status of clinical breast cancer tissues on tissue microarray (TMA) slides.

## Methods

### Tissue samples

FFPE MCF7 and Calu-3 xenograft tumors were used for the initial development and optimization of the HER2 gene-protein assay. MCF7 is a breast adenocarcinoma cell line in which the *HER2* gene is not amplified (average copy number = 2) and Calu-3 is a lung adenocarcinoma cell line in which *HER2* is amplified (average copy number = 30) [10]. Paraffin sections of the tumors were placed onto Superfrost Plus glass slides (Erie Scientific Company, Portsmouth, NH, USA) for analysis.

The performance of the HER2 gene-protein assay was examined using 189 breast cancer tissue cores on TMA slide sets provided by the National Cancer Center Hospital (NCCH), Tokyo, Japan. The breast cancer tissue samples were randomly selected from a tissue archive of samples acquired between 1991 and 1995. The protocol was approved by the NCCH Institutional Review Board. The TMA slides contained 36–41 tissue cores on a Matsunami Platinum coated glass slide (Matsunami Glass Ind., Ltd., Osaka, Japan). To orient the tissue cores and provide a positive control, two tissue cores from a selected *HER2*-amplified breast cancer case were included in each TMA block. The adhesion of the tissue cores onto the TMA slides was enhanced by baking the slides for 15 min at 65°C before each assay.

### IHC determination of HER2 protein expression

The FDA-approved HER2 IHC assay using PATHWAY HER-2/*neu* rabbit monoclonal antibody (clone 4B5; Ventana) was performed with *i*View DAB Detection Kit (Ventana) on a BenchMark XT automated staining system (Ventana). Briefly, the tissue sections were deparaffinized with EZ Prep (Ventana) at 75°C, heat pretreated in Cell Conditioning 1 (CC1; Ventana) using “standard cell conditioning” for antigen retrieval at 100°C, and then incubated with the anti-HER2 primary antibody for 32 min at 37°C after inactivation of the endogenous peroxidase with hydrogen peroxide for 4 min. They were then blocked using Endogenous Biotin Blocking Kit (Ventana), incubated with a biotinylated secondary antibody for 8 min, and incubated with a streptavidin-HRP conjugate for 8 min at 37°C. The immunolocalized HER2 protein was visualized using a copper-enhanced DAB reaction. The slides were counterstained with Hematoxylin II (Ventana) for 4 min and Bluing Reagent (Ventana) for 4 min and coverslips were applied by an automated coverslipper (Tissue-Tek Film Automated Coverslipper; Sakura Finetek Japan, Tokyo, Japan).

### Dual color BISH determination of *HER2*/CEN17 ratio

The FDA-approved dual color BISH assay (INFORM *HER2* Dual ISH DNA Probe Assay; Ventana) for *HER2* and CEN17 quantitation was also performed on the BenchMark XT using *HER2* and CEN17 probes labeled with 2,4-dinitrophenyl (DNP) and digoxigenin (DIG), respectively. Briefly, after the tissue cores were deparaffinized with EZ Prep at 75°C, they were subjected to three 12 min cycles of heat pretreatment at 90°C in EZ Prep-diluted Cell Conditioning 2 (CC2; Ventana) followed by protease digestion with ISH Protease 3 (Ventana) for 16 min at 37°C. The genomic DNA in tissue sections and the nick-translated *HER2* and CEN17 probes were co-denatured by heat treatment for 20 min at 80°C followed by a hybridization step for 6 h at 44°C. After three 8 min stringency washes were carried out in 2× SSC (Ventana) at 72°C, the *HER2* and CEN17 signals were detected using *ultra*View SISH DNP and *ultra*View Red ISH DIG Detection Kits (Ventana), respectively.

For *HER2* gene detection, the slides were incubated with a rabbit anti-DNP antibody for 20 min and then with a HRP-conjugated goat anti-rabbit antibody for 16 min at 37°C. The *HER2* BISH signal was detected as metallic silver deposits with silver acetate, hydroquinone, and hydrogen peroxide for 4 min at 37°C. For CEN17 detection, the slides were incubated with a mouse anti-DIG antibody for 20 min and then with an AP-conjugated goat anti-mouse antibody for 32 min at 37°C. The CEN17 BISH signal was developed as red dot staining with fast red and naphthol phosphate for 16 min. Finally, the slides were counterstained with Hematoxylin II for 8 min and with Bluing Reagent for 4 min. After the slides were rinsed and air-dried, coverslips were applied by the Tissue-Tek Film Coverslipper.

### Development and optimization of the HER2 gene-protein assay

The HER2 gene-protein assay was developed on the BenchMark XT using FFPE xenograft tumors and clinical breast cancer samples. The samples were stained under a variety of assay conditions to determine an optimum protocol needed to achieve HER2 protein, *HER2* gene, and CEN17 staining results comparable to those of the individual HER2 IHC and *HER2* & CEN17 BISH assays. Optimum signal detection in the HER2 gene-protein assay was achieved by performing the IHC procedure before the BISH procedure. Reagent lots were consistent for all TMA slides across all assays and all assays were completed within one week.

The breast cancer TMA slides were subjected to the final optimized HER2 gene-protein staining protocol after the paraffin-embedded tissue cores were deparaffinized with a Liquid Coverslip (Ventana)-primed EZ Prep method. For HER2 protein staining, the TMA slides were heat pretreated with CC1 standard cell conditioning at 100°C and endogenous peroxidase was inactivated by incubation with hydrogen peroxide for 4 min at 37°C. The tissue cores were incubated with the rabbit monoclonal anti-HER2 antibody for 32 min at 37°C and the endogenous biotin was blocked using Endogenous Biotin Blocking Kit. The slides were incubated with a biotinylated secondary antibody for 8 min and then with a HRP-conjugated streptavidin for 8 min at 37°C. A copper enhanced DAB reaction was used to visualize the HER2 protein.

For *HER2* gene & CEN17 staining, the TMA slides were subjected to three 12 min cycles of heat pretreatment in EZ Prep-diluted CC2 at 90°C and then to mild tissue digestion with ISH Protease 3 for 16 min at 37°C. The tissue samples were then hybridized with a cocktail of DNP-labeled *HER2* and DIG-labeled CEN17 probes at 44°C for 6 h after denaturing for 4 min at 80°C. HybClear blocking solution (Ventana), a hybridization buffer containing naphthol phosphate, was added to the probe cocktail to block the interaction between the DNP hapten on the *HER2* probe and the DAB deposit during hybridization. Three 8 min stringency washes were carried out in 2× SSC at 72°C.

For *HER2* gene detection, the tissue samples were incubated with a rabbit anti-DNP antibody for 20 min at 37°C followed by incubation with a HRP-conjugated goat anti-rabbit antibody for 24 min at 37°C. *HER2* BISH signal was developed for 8 min by the metallic silver deposit with silver acetate, hydroquinone, and hydrogen peroxide. For CEN17 detection, the slides were incubated with a mouse anti-DIG antibody for 20 min at 37°C followed by an AP-conjugated goat anti-mouse antibody incubation for 32 min at 37°C. CEN17 BISH signal was developed with a fast red and naphthol phosphate mixture for 12 min at 37°C. HER2 gene-protein slides were counterstained with Hematoxylin II for 8 min followed by Bluing Reagent for 4 min at 37°C. Air-dried slides were coverslipped with the film coverslipper.

### Evaluation of HER2 gene-protein assay performance

All tissue cores stained for the HER2 IHC, *HER2* & CEN17 BISH, and HER2 gene-protein assays were manually scored by three pathologists (MP, NW, PB). Two of the pathologists were experienced at scoring HER2 IHC and *HER2* & CEN17 BISH slides whereas the third was trained by reading the scoring guidelines immediately before scoring. In the HER2 IHC assay and HER2 gene-protein assay, tissue cores on TMA slides were scored for HER2 protein expression from 0 to 3+. In the *HER2* & CEN17 BISH assay and the HER2 gene-protein assay, *HER2* gene and CEN17 copy numbers were collected for calculating the ratio of *HER2*/CEN17 according to the scoring guideline.

All data analyses were conducted using SAS 9.2 software (SAS Institute Inc., Cary, North Carolina, USA). Continuous variables were summarized descriptively by sample size, mean, standard deviation (SD), median, minimum, and maximum. Discrete variables were summarized descriptively using counts and percentages. Assay results were treated as positive or negative as previously approved by the FDA: 1) HER2 IHC negative (0 or 1+) and positive (2+ or 3+) and 2) *HER2* & CEN17 BISH positive (*HER2*/CEN17 ratio ≥ 2.0) and negative (*HER2*/CEN17 ratio < 2.0).

The concordance of the results from pairs of readers and among pairs of tests was calculated according to the following (Table 1):

**Table 1:** Joint Frequency Table for HER2 Staining Status

		**Comparator Y**	
		***y*^+^**	***y*^−^**
Comparator X	*x*^+^	*a*	*b*
	*x*^−^	*c*	*d*

Where *x*^*+*^ is the number of X positive samples, *x*^−^ is the number of X negative samples, *y*^*+*^ is the number of Y positive samples, *y*^−^ is the number of Y negative samples, *a* is the number of *x*^+^*y*^+^ samples, *b* is the number of *x*^+^*y*^−^ samples, *c* is the number of *x*^−^*y*^+^ samples, and *d* is the number of *x*^−^*y*^−^ samples.

For analyses in which comparator X was a “test” group and comparator Y was a “reference” group (*e.g*., X was the HER2 gene-protein test and Y was the HER2 IHC test), concordance was assessed by calculating positive percent agreement (PPA) and negative percent agreement (NPA) among comparators according to the following formulas:

(1) $$\begin{array}{l} \text{PPA}=\left[a/\left(a+c\right)\right]\times 100\%\\ \text{NPA}=\left[d/\left(b+d\right)\right]\times 100\% \end{array}$$

When neither comparator was considered to be the reference (*e.g.*, X and Y were two different scorers), concordance was determined by calculating average positive agreement (APA) and average negative agreement (ANA) according to the following formulas [11]:

(2) $$\begin{array}{l} \text{APA}=\left[2a/\left(2a+b+c\right)\right]\times 100\%\\ \text{ANA}=\left[2d/\left(2d+b+c\right)\right]\times 100\% \end{array}$$

The overall concordance was calculated as the overall percent agreement (OPA) for all concordance analyses according to the formula:

(3) $$\text{OPA}=\left[\left(a+d\right)/\left(a+b+c+d\right)\right]\times 100\%$$

Two-sided 95% confidence intervals were calculated using the score method. Kappa coefficients were calculated for assay agreements for each analysis.

## Results

### Development of the HER2 gene-protein assay

**Figure 1:**
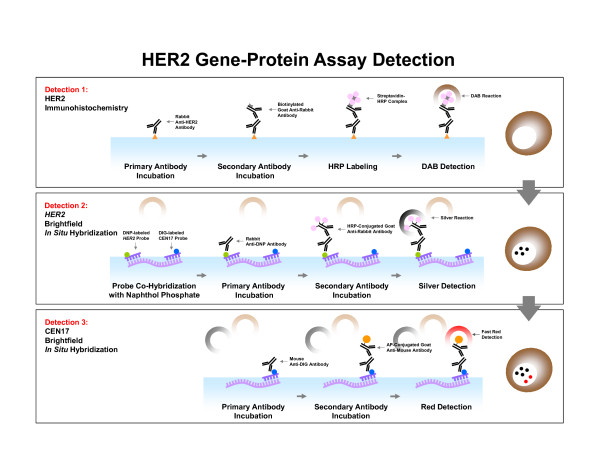
**Scheme for simultaneous visualization of human epidermal growth factor receptor 2 (HER2) protein, the* HER2 *gene, and the chromosome 17 centromere (CEN17) using a novel HER2 gene-protein assay.** First, HER2 protein is localized through immunohistochemical staining (IHC) with a rabbit monoclonal anti-HER2 antibody and a conventional 3,3′-diaminobenzidine (DAB)-based detection method. Then, the *HER2* gene and CEN17 are localized by brightfield *in situ* hybridization (BISH) with a cocktail of 2,4-dinitrophenyl (DNP)-labeled *HER2* probe and digoxigenin (DIG)-labeled CEN17 probe. The *HER2* gene and CEN17 signals are visualized with silver (a silver acetate, hydroquinone, and hydrogen peroxide reaction) and fast red (a fast red and naphthol phosphate reaction), respectively.

**Figure 2:**
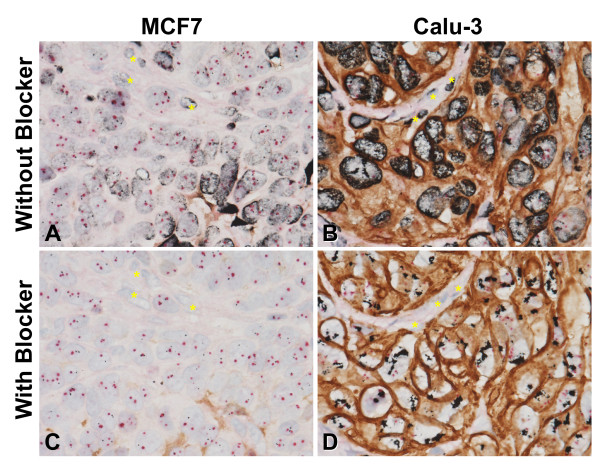
**Naphthol phosphate blocks silver background staining resulting from the* HER2 *& CEN17 BISH procedure.** Images show HER2 gene-protein staining results obtained without (**A**, **B**) or with naphthol phosphate (**C**, **D**) on formalin-fixed, paraffin-embedded (FFPE) HER2-negative MCF7 (**A**, **C**) and HER2-positive Calu-3 (**B**, **D**) xenograft tumors. In the absence of naphthol phosphate, non-specific silver deposition from the *HER2* BISH detection procedure obscures the BISH signals for the *HER2* gene and CEN17 targets (**A**, **B**) whereas the use of a BISH hybridization buffer containing naphthol phosphate eliminates the non-specific silver deposition (**C**, **D**). Some silver deposition was also seen in DAB staining (**A**). In the absence of naphthol phosphate, non-specific silver deposition occurred in mouse cells (yellow asterisks) (**A**, **B**) and mouse cells were confirmed without *HER2* and CEN17 BISH signals by using naphthol phosphate (**C**, **D**). 60×.

The optimized staining protocol for simultaneous visualization of HER2 protein, the *HER2* gene, and CEN17 on the same tissue is shown in Figure 1. As shown in the figure, the HER2 IHC detection is performed before the *HER2* & CEN17 BISH detection, as this sequence was found to be the most sensitive for HER2 protein detection (data not shown). The major technical difficulties encountered in combining the DAB-based HER2 IHC assay and the *HER2* & CEN17 BISH assay, which uses a DNP-labeled *HER2* probe, were high background staining of the nuclei and a change in the DAB staining appearance on FFPE tissue sections (Figure 2A, B). When the BISH step was carried out after the DAB-based IHC step, the DNP hapten on the *HER2* DNA probe bound non-specifically to nuclei and to some of the DAB staining, thus producing silver background staining during the BISH detection step for DNP-labeled DNA. The use of additional washing steps failed to reduce this artifactual background staining (data not shown), but the inclusion of naphthol phosphate in the hybridization buffer during hybridization was found to block DNP-DAB interaction, thereby eliminating the silver background staining (Figure 2C, D).

### The optimized HER2 gene-protein assay appropriately stains clinical breast-cancer tissue samples

**Figure 3:**
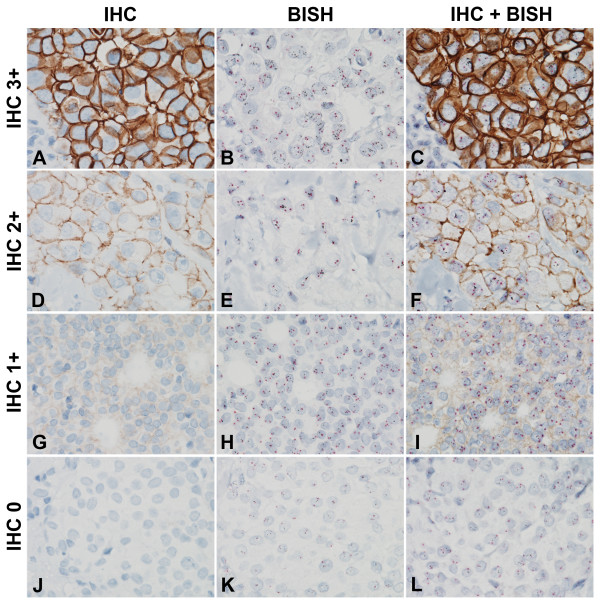
**The HER2 gene-protein assay yields appropriate staining of HER2 protein, the* HER2 *gene, and CEN17 in FFPE clinical breast cancer tissues.** Tissues with HER2 IHC scores of 3+ (**A**–**C**), 2+ (**D**–**F**), 1+ (**G**–**I**), and 0 (**J**–**L**) were subjected to HER2 IHC assay (**A**, **D**, **G**, **J**), *HER2* & CEN17 BISH assay (**B**, **E**, **H**, **K**), or the HER2 gene-protein assay (**C**, **F**, **I**, **L**) using tissue microarray slides. HER2 IHC assay yielded the expected HER2 protein staining (**A**, **D**, **G**, **J**) and the separate *HER2* & CEN17 BISH assay yielded the expected staining of the *HER2* gene (black dots) and CEN17 (red dots) (**B**, **E**, **H**, **K**). The combined assay yielded both the appropriate HER2 protein staining and the appropriate *HER2* gene and CEN17 staining (**C**, **F**, **I**, **L**). [All images 60×.].

Using a previously FDA-approved HER2 IHC assay, we identified clinical breast cancer tissue samples with HER2 expression scores of 3+ (Figure 3A, B, C), 2+ (Figure 3D, E, F), 1+ (Figure 3G, H, I), and 0 (Figure 3J, K, L). As expected, a previously FDA-approved *HER2* and CEN17 BISH assay resulted in labeling of the *HER2* gene and CEN17 targets in the same area of each tissue core as black and red dots, respectively (Figure 3B, E, H, K). The optimized HER2 gene-protein assay (with naphthol phosphate) stained the HER2 protein, *HER2* gene, and CEN17 targets as in the separate IHC and BISH assays (Figure 3C, F, I, J). Specifically, HER2 protein was visualized as complete membrane staining in the 3+ sample (Figure 3A, C), less complete membrane staining in the 2+ sample (Figure 3D, F), faint membrane staining in the 1+ sample (Figure 3G, I), and no staining in the 0 sample (Figure 3J, L), and the *HER2* gene and CEN17 targets appeared as black and red dots, respectively. Thus, the multiplex HER2 gene-protein assay co-localized the HER2 protein, *HER2* gene, and CEN17 targets appropriately.

### Performance of the optimized HER2 gene-protein Assay

**Figure 4:**
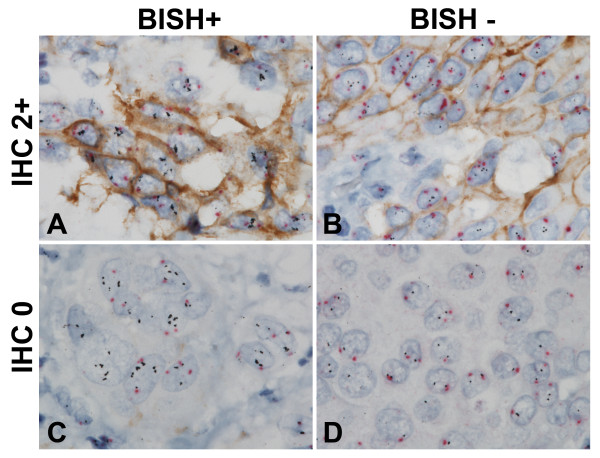
**Differentiation of* HER2 *gene status in HER2 protein equivocal (IHC 2+) or negative (IHC 0) breast cancer tissues using the HER2 gene-protein assay.** The HER2 gene-protein assay carried out on FFPE breast cancer tissue microarray slides was able to differentiate HER2 IHC 2+ cases that were *HER2* & CEN17 BISH positive (**A**) and negative (**B**). It also distinguished HER2 IHC 0 cases that were *HER2* & CEN17 BISH positive (**C**) and negative (**D**). [All images 100**×**.].

The optimized HER2 gene-protein assay in determining both HER2 protein (IHC) and *HER2* gene (BISH) status simultaneously demonstrated the usefulness of the assay on equivocal (2+) and negative (0) HER2 IHC samples of different *HER2* ISH statuses (Figure 4). It readily distinguished the *HER2* ISH positive samples from the *HER2* ISH negative samples in both the IHC equivocal (Figure 4A and B, respectively) and the IHC-negative cases (Figure 4C and D, respectively).

**Figure 5:**
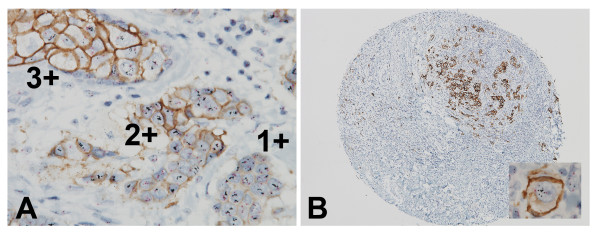
**Results of HER2 gene-protein staining of FFPE breast cancer tissues exhibiting heterogeneity of HER2 positive tumor cell populations or isolated tumor cell populations.** (**A**) The HER2 gene-protein assay demonstrated the heterogeneity of HER2 positive tumor cell populations in FFPE breast cancer tissues. In the sample shown, cell populations with HER2 IHC scores of 3+, 2+, and 1+ neighbor each other and all tumor populations present amplified *HER2* gene. However, the HER2 IHC 3+ tumor cell population contains dispersed *HER2* gene copies while the HER2 IHC 2+ and 1+ population contains clustered *HER2* gene copies [40×]. (**B**) The HER2 gene-protein assay clearly visualized small groups of HER2 IHC 3+ breast cancer cells [4×]. The insert shows an isolated individual HER2 IHC positive tumor cell with *HER2* gene amplification [100×].

For one breast cancer tissue core, the HER2 gene-protein assay revealed the heterogeneity of HER2 protein status with HER2 IHC 3+, 2+, and 1+ cell populations visible within the same area (Figure 5A). It also showed that the HER2 IHC 3+ cell population contained dispersed *HER2* gene copies while the HER2 IHC 2+ and 1+ cell populations contained clustered *HER2* gene copies. The HER2 gene-protein assay also detected the scattered HER2 positive cell populations within a breast cancer tissue core (Figure 5B). Isolated HER2 IHC positive and *HER2* & CEN17 BISH positive tumor cells surrounded by the interstitial tissue were clearly visible (Figure 5B, insert).

Because six of the 189 breast tissue cores examined contained no tumor cells (Table 2), all statistical analyses were based on the 183 remaining tissue cores with tumor cells. All of these cores yielded evaluable IHC results (from both the single and combined assays) for each of the three readers in this study (Table 2). For the BISH results, the percentage of evaluable cores ranged from 90.7% (Reader A, *HER2* & CEN17 BISH) to 98.4% (Reader C, HER2 gene-protein assay). The HER2 gene-protein assay consistently yielded a higher number of evaluable cores than the *HER2* & CEN17 BISH assay, although most of these were evaluable (Table 2). The superiority of the HER2 gene-protein assay over the *HER2* & CEN17 BISH assay in yielding BISH results ranged by reader from approximately 7% to 13%.

**Table 2:** Disposition of study cases

**Parameter**	**Count**	**Reader A**	**Reader B**	**Reader C**
	***n***	***n*** (%)	***n*** (%)	***n*** (%)
Total number of tissue cores	189			
Number of tissue cores without tumor	6			
Number of cores with tumor	183			
Number of cores with evaluable gene-protein IHC results		183 (100.0)	183 (100.0)	183 (100.0)
Number of cores with evaluable single IHC results		183 (100.0)	183 (100.0)	183 (100.0)
Number of cores with evaluable gene-protein BISH results		179 (97.8)	179 (97.8)	180 (98.4)
Number of cores with evaluable single BISH results		166 (90.7)	172 (94.0)	173 (94.5)

*IHC*: immunohistochemistry; *BISH*: brightfield *in situ* hybridization.

The HER2 IHC score distributions for the HER2 IHC assay relative to the HER2 gene-protein assay were comparable for each reader and the distributions of positive *vs.* negative HER2 IHC assessments were consistent from assay to assay and also from reader to reader (Table 3). For both types of HER2 IHC and for all readers, approximately 30% of HER2 IHC assessments were positive and approximately 70% were negative (Table 3). For each reader, the average *HER2* gene count, CEN17 count, and *HER2*/CEN17 ratio were very similar between the *HER2* & CEN17 BISH and the HER2 gene-protein assay, and the *HER2* gene amplification status was comparable between the two assays (Table 4). The distribution of amplified *vs.* non-amplified *HER2* & CEN17 BISH assessments was consistent from assay to assay and also from reader to reader (Table 4). For both types of *HER2* & CEN17 BISH and for all readers, approximately 25–27% of samples were *HER2* amplified and approximately 67–72% were non-amplified when unevaluable tissue cores were included in the calculation of percentage (Table 4).

**Table 3:** IHC descriptive statistics

	**Reader A**		**Reader B**		**Reader C**	
	**Gene-protein IHC (N = 183)**	**Single IHC (N = 183)**	**Gene-protein IHC (N = 183)**	**Single IHC (N = 183)**	**Gene-protein IHC (N = 183)**	**Single IHC (N = 183)**
**IHC score**, n (%)						
0	84 (45.9)	90 (49.2)	99 (54.1)	109 (59.6)	110 (60.1)	115 (62.8)
1+	47 (25.7)	43 (23.5)	34 (18.6)	26 (14.2)	18 (9.8)	14 (7.7)
2+	10 (5.5)	8 (4.4)	7 (3.8)	6 (3.3)	8 (4.4)	10 (5.5)
3+	42 (23.0)	42 (23.0)	43 (23.5)	42 (23.0)	47 (25.7)	44 (24.0)
**IHC assessment**, n (%)						
Positive	52 (28.4)	50 (27.3)	50 (27.3)	48 (26.2)	55 (30.1)	54 (29.5)
Negative	131 (71.6)	133 (72.7)	133 (72.7)	135 (73.8)	128 (69.9)	129 (70.5)

*IHC*: immunohistochemistry.

**Table 4:** BISH descriptive statistics

	**Reader A**		**Reader B**		**Reader C**	
	**Gene-protein BISH (N = 183)**	**Single BISH (N = 183)**	**Gene-protein BISH (N = 183)**	**Single BISH (N = 183)**	**Gene-protein BISH (N = 183)**	**Single BISH (N = 183)**
***HER2*mean**						
n	179	166	179	172	180	173
Mean (SD)	4.273 (4.208)	4.643 (4.567)	5.199 (6.280)	5.452 (6.693)	3.333 (2.851)	3.176 (2.362)
Median	2.200	2.225	1.900	1.900	2.000	1.950
Min, max	1.00, 21.10	1.00, 22.60	1.00, 40.20	1.00, 40.40	1.00, 16.25	1.20, 11.10
**CEN17 mean**						
n	179	166	179	172	180	173
Mean (SD)	1.803 (0.496)	1.868 (0.501)	1.566 (0.356)	1.540 (0.339)	1.606 (0.335)	1.613 (0.296)
Median	1.650	1.750	1.500	1.450	1.550	1.550
Min, max	1.00, 3.50	1.00, 3.95	1.00, 2.80	1.00, 2.95	1.05, 2.60	1.00, 2.65
***HER2*/CEN17 ratio**						
n	179	166	179	172	180	173
Mean (SD)	2.485 (2.613)	2.521 (2.533)	3.344 (3.952)	3.637 (4.620)	2.100 (1.802)	2.004 (1.564)
Median	1.170	1.190	1.310	1.355	1.255	1.240
Min, max	0.67, 10.82	0.76, 11.02	0.67, 19.14	0.60, 26.06	0.74, 8.46	0.82, 9.25
**Amplification status**, n (%)						
Amplified	46 (25.1)	46 (25.1)	49 (26.8)	50 (27.3)	47 (25.7)	43 (23.5)
Non-amplified	133 (72.7)	120 (65.6)	130 (71.0)	122 (66.7)	133 (72.7)	130 (71.0)
N/A	4 (2.2)	17 (9.3)	4 (2.2)	11 (6.0)	3 (1.6)	10 (5.5)

*BISH*: brightfield *in situ* hybridization; *HER2*: human epidermal growth factor receptor 2; *CEN17*: chromosome 17 centromere.

In concordance analyses, the HER2 IHC assay was taken as the reference assay for the HER2 protein expression results of the HER2 gene-protein assay (Table 5). The IHC positive agreement ranged from 97.9% (Reader B) to 100% (Readers A and C) and the IHC negative agreement ranged from 97.8% (Reader B) to 99.2% (Reader C). The overall agreement ranged from 97.8% (Reader B) to 99.5% (Reader C) and the kappa coefficient ranged from 0.94 (Reader B) to 0.99 (Reader C). These excellent agreement rates indicate that HER2 status diagnosis based on the HER2 IHC part of the combined HER2 gene-protein assay is essentially equivalent to that based on the individual HER2 IHC assay.

**Table 5:** **Gene-protein IHC (comparator)*vs.*single IHC (reference) agreement analyses**

**Single IHC**				
	**Gene-protein IHC**	**Positive**	**Negative**	**Total**
**Reader A**	Positive	50	2	52
	Negative	0	131	131
	Total	50	133	183
	PPA: n/N (%) (95% CI)	50/50 (100.0) (92.9–100.0)		
	NPA: n/N (%) (95% CI)	131/133 (98.5) (94.7–99.6)		
	OPA: n/N (%) (95% CI)	181/183 (98.9) (96.1–99.7)		
	Kappa coefficient (95% CI)	0.97 (0.94–1.00)		
**Reader B**	Positive	47	3	50
	Negative	1	132	133
	Total	48	135	183
	PPA: n/N (%) (95% CI)	47/48 (97.9) (89.1–99.6)		
	NPA: n/N (%) (95% CI)	132/135 (97.8) (93.7–99.2)		
	OPA: n/N (%) (95% CI)	179/183 (97.8) (94.5–99.1)		
	Kappa coefficient (95% CI)	0.94 (0.89–1.00)		
**Reader C**	Positive	54	1	55
	Negative	0	128	128
	Total	54	129	183
	PPA: n/N (%) (95% CI)	54/54 (100.0) (93.4–100.0)		
	NPA: n/N (%) (95% CI)	128/129 (99.2) (95.7–99.9)		
	OPA: n/N (%) (95% CI)	182/183 (99.5) (97.0–99.9)		
	Kappa coefficient (95% CI)	0.99 (0.96–1.00)		

*IHC*: immunohistochemistry; *PPA*: positive percent agreement; *NPA*: negative percent agreement; *OPA*: overall percent agreement.

In concordance analyses, the *HER2* & CEN17 BISH assay was taken as the reference assay for the *HER2* & CEN17 BISH results of the HER2 gene-protein assay (Table 6). The *HER2* & CEN17 BISH positive agreement ranged from 94% (Reader B) to 95.6% (Reader A) and the BISH negative agreement ranged from 96.2% (Reader C) to 99.2% (Reader B). The overall percent agreement ranged from 96% (Reader C) to 97.7% (Reader B) and the kappa coefficient ranged from 0.89 (Reader C) to 0.94 (Readers A and B), indicating that the *HER2* diagnosis based on the *HER2* & CEN17 BISH part of the HER2 gene-protein assay is essentially equivalent to that based on the *HER2* & CEN17 BISH only assay.

**Table 6:** **Gene-protein BISH (comparator)*vs.*single BISH (reference) agreement analyses**

**Single BISH**				
	**Gene-protein BISH**	**Amplified**	**Non-amplified**	**Total**
**Reader A**	Amplified	43	2	45
	Non-amplified	2	118	120
	Total	45	120	165
	PPA: n/N (%) (95% CI)	43/45 (95.6) (85.2–98.8)		
	NPA: n/N (%) (95% CI)	118/120 (98.3) (94.1–99.5)		
	OPA: n/N (%) (95% CI)	161/165 (97.6) (93.9–99.1)		
	Kappa coefficient (95% CI)	0.94 (0.88–1.00)		
**Reader B**	Amplified	47	1	48
	Non-amplified	3	121	124
	Total	50	122	172
	PPA: n/N (%) (95% CI)	47/50 (94.0) (83.8–97.9)		
	NPA: n/N (%) (95% CI)	121/122 (99.2) (95.5–99.9)		
	OPA: n/N (%) (95% CI)	168/172 (97.7) (94.2–99.1)		
	Kappa coefficient (95% CI)	0.94 (0.89–1.00)		
**Reader C**	Amplified	41	5	46
	Non-amplified	2	125	127
	Total	43	130	173
	PPA: n/N (%) (95% CI)	41/43 (95.3) (84.5–98.7)		
	NPA: n/N (%) (95% CI)	125/130 (96.2) (91.3–98.3)		
	OPA: n/N (%) (95% CI)	166/173 (96.0) (91.9–98.0)		
	Kappa coefficient (95% CI)	0.89 (0.82–0.97)		

*BISH*: brightfield *in situ* hybridization; *PPA*: positive percent agreement; *NPA*: negative percent agreement; *OPA*: overall percent agreement.

Analyses of the combined HER2 IHC and *HER2* & CEN17 BISH data from each reader for the individual IHC and BISH assays and the gene-protein assay are shown in Table 7. For analyses using the HER2 gene-protein assay, the status of a tissue core was defined as HER2 positive if either the HER2 protein status was positive or the *HER2* gene status was amplified; otherwise, it was defined as negative. This algorithm is consistent with clinical practice, in which a patient is treated as HER2 positive if either assay shows an amplified HER2 status. The results of the individual *HER2* & CEN17 BISH and HER2 IHC assays were similarly combined to yield the overall HER2 status that served as the reference for the combined assay. The HER2 positive agreement ranged from 94.3% (Reader B) to 98% (Reader A) and the HER2 negative agreement ranged from 97.5% (Reader C) to 98.3% (Reader B). The overall agreement ranged from 97.1% (Readers B and C) to 98.2% (Reader A) and the kappa coefficient ranged from 0.93 (Readers B and C) to 0.96 (Reader A), indicating that HER2 diagnosis based on the BISH and IHC results of the HER2 gene-protein assay is essentially equivalent to that based on the separate *HER2* & CEN17 BISH and HER2 IHC assays.

**Table 7:** **Gene-protein IHC/BISH (comparator)*vs.*single IHC/BISH (reference) agreement analyses**

**Single IHC/BISH assay**				
	**Gene-protein IHC/BISH**	**Positive**	**Negative**	**Total**
**Reader A**	Positive	50	2	52
	Negative	1	112	113
	Total	51	114	165
	PPA: n/N (%) (95% CI)	50/51 (98.0) (89.7–99.7)		
	NPA: n/N (%) (95% CI)	112/114 (98.2) (93.8–99.5)		
	OPA: n/N (%) (95% CI)	162/165 (98.2) (94.8–99.4)		
	Kappa coefficient (95% CI)	0.96 (0.91–1.00)		
**Reader B**	Positive	50	2	52
	Negative	3	117	120
	Total	53	119	172
	PPA: n/N (%) (95% CI)	50/53 (94.3) (84.6–98.1)		
	NPA: n/N (%) (95% CI)	117/119 (98.3) (94.1–99.5)		
	OPA: n/N (%) (95% CI)	167/172 (97.1) (93.4–98.8)		
	Kappa coefficient (95% CI)	0.93 (0.87–0.99)		
**Reader C**	Positive	52	3	55
	Negative	2	116	118
	Total	54	119	173
	PPA: n/N (%) (95% CI)	52/54 (96.3) (87.5–99.0)		
	NPA: n/N (%) (95% CI)	116/119 (97.5) (92.8–99.1)		
	OPA: n/N (%) (95% CI)	168/173 (97.1) (93.4–98.8)		
	Kappa coefficient (95% CI)	0.93 (0.88–0.99)		

*IHC*: immunohistochemistry; *BISH*: brightfield *in situ* hybridization; *PPA*: positive percent agreement; *NPA*: negative percent agreement; *OPA*: overall percent agreement.

Note: IHC/BISH status: positive = IHC is positive or BISH is amplified; negative = IHC is negative and BISH is non-amplified.

Analyses of the agreement among readers were performed separately for the HER2 IHC protein statuses from the single IHC assay and the gene-protein assay, for the *HER2* BISH gene statuses from the single assay and the gene-protein assay, for the combined HER2 IHC and *HER2* & CEN17 BISH results from two separate *HER2* & CEN17 BISH and HER2 IHC assays and the gene-protein assay (Table 8). Each of the three possible reader pairs was assessed for each assay. Because no individual reader was considered the reference, reader-to-reader agreement was assessed by calculating APA and ANA. Of the 24 calculated reader-to-reader agreement rates, 17 were greater than 95%, 5 were between 90% and 95%, and 2 were close to 90%, indicating excellent inter-reader agreement on all assays. The APA and ANA for all reader pairs based on HER2 IHC results, *HER2* & CEN17 BISH results, and combined HER2 IHC and *HER2* & CEN17 BISH results were always higher in the HER2 gene-protein assay than in two separate HER2 IHC and *HER2* & CEN17 BISH assays and the difference ranged from 0.1–5.6% (Table 9). Thus, when the *HER2* & CEN17 BISH and HER2 IHC assays were combined into a single test as the gene-protein assay, inter-reader agreement consistently improved over two separate HER2 IHC and *HER2* & CEN17 BISH assays.

**Table 8:** Summary of inter-reader agreement analyses

**Assay**	**Minimum APA**	**Maximum APA**	**Minimum ANA**	**Maximum ANA**
Gene-protein IHC	95.2%	97.2%	98.1%	98.8%
Single IHC	92.2%	96.2%	97.0%	98.5%
Gene-protein BISH	93.5%	97.9%	97.7%	99.2%
Single BISH	88.2%	93.8%	95.5%	97.5%
Gene-protein IHC/BISH	91.7%	95.2%	96.4%	98.0%
Single IHC/BISH	89.7%	94.3%	95.2%	97.3%

*APA*: average positive agreement; *ANA*: average negative agreement; *IHC*: immunohistochemistry; *BISH*: brightfield *in situ* hybridization.

**Table 9:** Inter-reader agreement by reader pair and assay

**Reader Pair**	**Assay**	**APA**	**ANA**
A *vs.* B	Gene-protein IHC	96.1	98.5
	Single IHC	93.9	97.8
	Gene-protein BISH	97.9	99.2
	Single BISH	93.8	97.5
	Gene-protein IHC/BISH	95.2	98.0
	Single IHC/BISH	93.3	96.9
A *vs.* C	Gene-protein IHC	97.2	98.8
	Single IHC	96.2	98.5
	Gene-protein ISH	93.5	97.7
	Single BISH	92.1	97.0
	Gene-protein IHC/BISH	94.4	97.6
	Single IHC/BISH	94.3	97.3
B *vs.* C	Gene-protein IHC	95.2	98.1
	Single IHC	92.2	97.0
	Gene-protein BISH	93.8	97.7
	Single BISH	88.2	95.5
	Gene-protein IHC/BISH	91.7	96.4
	Single IHC/BISH	89.7	95.2

*APA*: average positive agreement; *ANA*: average negative agreement; *IHC*: immunohistochemistry; *BISH*: brightfield *in situ* hybridization.

## Discussion

To avoid subjecting breast cancer patients to unnecessary financial burden and significant side effects, the selection of those most likely to response to HER2-directed therapy must be accurate. Our original motivation for developing the tricolor HER2 gene-protein assay was to deliver a tissue-based HER2 test that is more accurate than the separate HER2 IHC and *HER2* ISH assays. Because HER2 IHC assays are technically easier to perform than *HER2* FISH assays, 80% of newly diagnosed breast cancer cases in the US are analyzed for HER2 status using HER2 IHC [12]. However, the technical issues that can complicate HER2 IHC assays, such as unstandardized antigen retrieval protocols and multiple antibody clones, have led to the recommendation of *HER2* ISH as the first-line assay for HER2 status assessment [13]. Tissue quality for *HER2* ISH assays can be assessed using the ISH signals in normal cells surrounding the tumor cells as internal controls. In contrast, assessment of tissue quality for HER2 IHC assays is difficult; because there is no proven internal control, false negatives can result [14].

A 2007 report of the American Society of Clinical Oncology-College of American Pathologists (ASCO-CAP) concluded that 20% of HER2 assays performed in the field were not accurate and established guidelines to improve the accuracy of HER2 testing in breast cancer [2]. However, a 2008 follow up study using survey results from 757 laboratories indicated that substantial gaps remained in assay validation [15]. Lee *et al.*[1] also reported that only 15% (7/46) of reported studies of the concordance between HER2 IHC and *HER2* FISH results achieved the ASCO-CAP guideline of 95% or greater concordance.

Breast tumor heterogeneity is a major cause of discordance between HER2 IHC and *HER2* FISH assay results [16,17] and approximately 5-30% of HER2 positive breast cancer cases exhibit intratumoral genetic heterogeneity [18]. Subtle *HER2* genetic heterogeneity of tumor cells has been reported among equivocal cases [17,19]. An alternative method for determining HER2 status from FFPE breast cancer samples based on the quantitative reverse transcription-polymerase chain reaction (qRT-PCR) has been proposed, but has not been approved by the FDA. Based on a recent publication comparing the performance of HER2 qRT-PCR-based testing with that of the FDA-approved HER2 IHC and *HER2* FISH methods [20], Ignatiadis and Sotirious have raised concerns about the use of HER2 qRT-PCR for clinical diagnostics [21]. The HER2 qRT-PCR method failed to detect equivocal cases and produced false negative results. Therefore, the need for a better assay to assess HER2 status in breast cancer, particularly in equivocal cases and in cases with tumor heterogeneity, remains.

With an incidence of approximately 4%, HER2 false negative (IHC negative and FISH positive) and false positive (IHC positive and FISH negative) results cannot be ignored [1,17]. In one study, 9.7% (174/1787) of breast cancer patients were HER2 false positive cases, but they still benefited from adjuvant trastuzumab therapy [22]. In another study, lapatinib therapy had significant positive effects in FISH positive breast cancer patients whose IHC tests had been 0, 1+, or 2+ [13]. Thus, the detection of both false negative and false positive HER2 breast cancer cases is important.

HER2 IHC assays are effective methods for detecting tumor heterogeneity and equivocal cases based on HER2 protein staining under a light microscope, but these assays are semi-quantitative and subjective. Thus, additional quantitative gene analysis is required for equivocal cases using a *HER2* ISH assay. HER2 false negative cases will be missed if only HER2 IHC is applied while HER2 false positive cases will be missed if only *HER2* ISH method is utilized. Thus, the optimum HER2 testing protocol uses both HER2 IHC and *HER2* ISH assays [23].

To overcome the weaknesses of current HER2 tests, we have successfully developed an automated brightfield tricolor gene-protein assay for the detection of HER2 protein, the *HER2* gene, and CEN17. The novel aspect of the assay is the use of a blocker to prevent background staining caused by the binding of the DNP hapten of the *HER2* probe to tissue sections after they have been processed through a DAB-based IHC assay. Although three research groups have previously reported the technical achievement of combining HER2 IHC and single color brightfield *HER2* ISH to co-visualize HER2 protein and the *HER2* gene on FFPE breast cancer tissue sections, all of these combined assays required several manual steps for the ISH procedure.

The HER2 gene-protein assay described herein is a significant improvement in the field because: 1) it demonstrates tricolor co-localization of the HER2 protein, *HER2* gene, and CEN17 targets on well-preserved breast cancer tissue sections and 2) it automates the entire protocol of a gene-protein assay from deparaffinization to counterstaining. Extensive analyses of the findings of three pathologists with different levels of HER2 test scoring experience for the combination HER2 gene-protein assay relative to those of the single HER2 IHC and *HER2* & CEN17 BISH assays revealed excellent concordance. The statistical analysis suggests that the HER2 gene-protein assay is a robust and reliable assay and provides advantages over single HER2 IHC and *HER2* & CEN17 BISH assays.

Among the technical challenges we faced in developing the HER2 gene-protein assay was identifying an appropriate multicolor scheme. Previously, Downs-Kelly [7] and Ni *et al.*[8] used AP-based fast red staining of HER2 protein followed by HRP-based silver or DAB staining of *HER2* gene, respectively. We evaluated AP-based fast blue detection of HER2 IHC followed by the *HER2* BISH assay to obtain a tricolor detection scheme in which HER2 protein was blue, the *HER2* gene was black, and CEN17 was red (data not shown), but this scheme proved to be less than optimal; the pathologists had difficulty scoring weak HER2 IHC staining because the fast blue AP-based IHC staining was less crisp and because both fast blue and hematoxylin counterstain are blue. Therefore, because most pathologists are accustomed to scoring DAB-based IHC detection for HER2 protein, we investigated a detection scheme using a combination of conventional DAB-based detection of HER2 protein and BISH detection of *HER2* and CEN17 targets. The sequence of HER2 IHC and *HER2* & CEN17 BISH staining was also evaluated to optimize HER2 protein staining. As Reisenbichler *et al.*[9] previously noted, we observed weaker HER2 protein staining when the HER2 IHC portion of the assay was performed after the *HER2* & CEN17 BISH portion of the assay (data not shown). They compensated for the weaker HER2 IHC staining by increasing the anti-HER2 antibody incubation time from 30 min to 45 min. In contrast, we determined that the HER2 IHC steps should be performed first to maintain HER2 IHC staining quality, particularly in cases with low expressed HER2 protein.

Reisenbichler *et al.*[9] also reported that they could not obtain *HER2* CISH signals when the CISH assay was performed after HER2 IHC using DAB detection. We encountered a similar obstacle during the development of our HER2 gene-protein assay, but primarily for CEN17 BISH detection. We found that a longer protease digestion time or a higher protease concentration was required to obtain a consistent CEN17 BISH signal with difficult tissue samples to stain for CEN17 signals. As we have demonstrated in this report, our optimized *HER2* & CEN17 BISH assay provided successful visualization of the *HER2* gene and CEN17 targets after DAB-based HER2 IHC.

Another major issue encountered during assay development was a high level of silver background staining from the silver-based *HER2* BISH detection. The silver background staining was observed mainly in the nuclei and also some background staining was seen with DAB staining. It did not occur when the DNP-labeled *HER2* probe was omitted from the assay (data not shown). Also, omission of the DAB chromogen and hydrogen peroxide from the IHC procedure prevented silver background staining from silver-based *HER2* BISH detection (data not shown). Therefore, we hypothesized that there was an interaction between DAB and the DNP hapten and the BISH detection for the DNP hapten was responsible for the high levels of silver background staining. Because extra washing after the HER2 IHC did not eliminate the silver background staining (data not shown), we concluded that the DAB molecules were covalently bound to the nuclear DNA.

It is well established that DAB is a carcinogen and that carcinogenic agents bind to DNA. Oxidative intermediates of the DAB analogue benzidine have been shown to form covalent bonds to DNA, thereby localizing DAB in the cell nucleus [24,25]. During the development of the HER2 gene-protein assay, the DNP-labeled probes appeared to be binding to the peroxidase deposited DAB. The exact mechanism of this interaction is unknown, but electron-rich aromatic compounds (such as DAB) and electron-deficient aromatic compounds (such as DNP) are known to form aromatic pi-stacks and/or charge transfer complexes [26]. Therefore, we speculated that another aromatic molecule present in excess during hybridization would act as a competitor for this non-covalent attraction, analogous to the use of protein blockers in protein immunodetection to prevent non-specific protein binding. After testing several compounds with various electronic and aqueous solubility properties (data not shown), we identified naphthol phosphate as a suitable blocker for use during hybridization with DNP-labeled probes.

A HER2 gene-protein assay could be developed by combining two darkfield assays, namely HER2 fluorescence IHC and *HER2* FISH assays. However, the current brightfield HER2 gene-protein assay offers several advantages over a darkfield gene-protein assay: 1) the ability to simultaneously observe the HER2 protein, *HER2*, and CEN17 targets in the context of tissue morphology; 2) the use of an established scoring system for DAB-based HER2 IHC assays; 3) the use of a regular light microscope for slide observations, negating the need for a darkroom; 4) full automation, which is optimal for reproducibility; and 5) permanent preservation of both the IHC and ISH signals. A HER2 gene-protein assay could also be developed using DAB-based HER2 IHC and *HER2* FISH assays. This assay would have fewer disadvantages than the gene-protein assay using fluorescence IHC, but would still require an expensive fluorescence microscope and a darkroom. In addition, long term preservation of the FISH signal would be difficult and no completely automated protocol would be available for this assay.

## Conclusions

We have developed a robust automated procedure for the simultaneous visualization of HER2 protein, the *HER2* gene, and CEN17 in FFPE xenograft tumor tissue sections and have demonstrated its accuracy in the analysis of FFPE breast cancer TMA slides. The successful multiplexing of two FDA-approved HER2 IHC and *HER2* & CEN17 BISH assays was achieved by the inclusion of naphthol phosphate in the hybridization buffer to eliminate the silver background staining resulting from the *HER2* BISH detection. This HER2 gene-protein assay demonstrated the heterogeneity of HER2 protein expression in breast cancer cell populations and the simultaneous detection of HER2 protein, the *HER2* gene, and CEN17 allowed differentiation of HER2 IHC 2+ cases to *HER2* & CEN17 BISH positive or negative. Furthermore, it correctly identified cases yielding false negatives in HER2 IHC tests as *HER2* & CEN17 BISH positive. This new method for brightfield tricolor detection of HER2 protein, the *HER2* gene, and CEN17 might be useful in more accurately assessing the HER2 status of breast cancer patients, particularly in equivocal cases or cases with heterogeneous tumors. The HER2 gene-protein assay might also be useful in gastric cancer, which often exhibits tumor heterogeneity. Furthermore, this strategy for gene-protein detection assays could be applied to other cancer biomarkers, such as EGFR and *met* proto-oncogene.

## Abbreviations

ANA: Average negative agreement; AP: Alkaline phosphatase; APA: Average positive agreement; ASCO: American Society of Clinical Oncology; BISH: Brightfield in situ hybridization; CAP: College of American Pathologists; CEN17: Chromosome 17 centromere; CISH: Chromogenic in situ hybridization; DAB: 3,3′-diaminobenzidine; DIG: Digoxigenin; DNP: 2,4-dinitrophenyl; EGFR: Epidermal growth factor receptor; FDA: United States Food and Drug Administration; FFPE: Formalin-fixed paraffin-embedded; FISH: Fluorescence in situ hybridization; HER2: Human epidermal growth factor receptor 2; HRP: Horseradish peroxidase; IHC: Immunohistochemistry; ISH: In situ hybridization; NPA: Negative percent agreement; OPA: Overall percent agreement; PPA: Positive percent agreement; qRT-PCR: Quantitative reverse transcription-polymerase chain reaction; TMA: Tissue microarray.

## Competing interests

HN, MP, BK, NW, PB, SS, IB, JRM, CB, and TM are employed by Ventana Medical Systems, Inc. HT received a grant in support of this research from Ventana Medical Systems, Inc.

## Authors’ contributions

HN and TMG developed the HER2 gene-protein assay, performed feasibility studies, stained the clinical samples, and prepared the manuscript draft and image data. BK, HN, CB, and TMG identified the blocker for HER2 gene-protein assay. Pathologists MP, NW, PB, SS, and HT consulted in assay development and/or scored samples for assay development and validation. IB and JRM conducted all statistical analyses. All authors provided intellectual input for the study and approved the final manuscript.
